# Chemical group-dependent plasma polymerisation preferentially directs adipose stem cell differentiation towards osteogenic or chondrogenic lineages

**DOI:** 10.1016/j.actbio.2016.12.016

**Published:** 2017-03-01

**Authors:** M.F. Griffin, A. Ibrahim, A.M. Seifalian, P.E.M. Butler, D.M. Kalaskar, P. Ferretti

**Affiliations:** a UCL Centre for Nanotechnology and Regenerative Medicine, Division of Surgery & Interventional Science, University College London, London, United Kingdom; b Royal Free London NHS Foundation Trust Hospital, London, United Kingdom; c Stem Cells and Regenerative Medicine Section, UCL Great Ormond Street Institute of Child Health, University College London, London WC1N 1EH, United Kingdom

**Keywords:** Adipose stem cell, Chondrogenesis, Osteogenesis, Surface chemistry, Plasma polymerisation, Carboxyl, Amine

## Abstract

Human adipose derived stem cells (ADSCs) are being explored for the repair of craniofacial defects due to their multi-differentiation potential and ease of isolation and expansion. Crucial to using ADSCs for craniofacial repair is the availability of materials with appropriate biomechanical properties that can support their differentiation into bone and cartilage. We tested the hypothesis that different modifications of chemical groups on the surface of a nanocomposite polymer could increase human ADSC adhesion and selectively enhance their osteogenic and chondrogenic differentiation. We show that the COOH modification significantly promoted initial cell adhesion and proliferation over 14 days compared to NH_2_ surfaces. Expression of focal adhesion kinase and vinculin was enhanced after plasma surface polymerisation at 24 h. The COOH modification significantly enhanced chondrogenic differentiation as indicated by up-regulation of aggrecan and collagen II transcripts. In contrast, NH_2_ group functionalised scaffolds promoted osteogenic differentiation with significantly enhanced expression of collagen I, alkaline phosphatase and osteocalcin both at the gene and protein level. Finally, chorioallantoic membrane grafting demonstrated that both NH_2_ and COOH functionalised scaffolds seeded with ADSCs were biocompatible and supported vessel ingrowth apparently to a greater degree than unmodified scaffolds. In summary, our study shows the ability to direct ADSC chondrogenic and osteogenic differentiation by deposition of different chemical groups through plasma surface polymerisation. Hence this approach could be used to selectively enhance bone or cartilage formation before implantation *in vivo* to repair skeletal defects.

**Statement of Significance:**

Human adipose derived stem cells (hADSCs) are an exciting stem cell source for regenerative medicine due to their plentiful supply and ease of isolation. However, the optimal environmental cues to direct stem cells towards certain lineages change have to has not been identified. We have shown that by modifying the surface of the scaffold with specific chemical groups using plasma surface polymerisation techniques we can control ADSCs differentiation. This study shows that ADSCs can be differentiated towards osteogenic and chondrogenic lineages on amine (NH_2_) and carboxyl (COOH) modified scaffolds respectively. Plasma polymerisation can be easily applied to other biomaterial surfaces to direct stem cell differentiation for the regeneration of bone and cartilage.

## Graphical abstract

**Figure:**
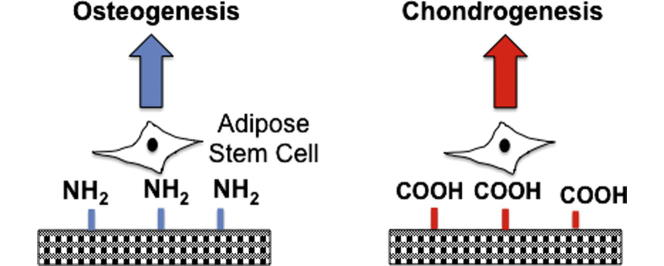

## 1 Introduction

Craniofacial defects caused by congenital deformities, cancer, trauma or burns remain one of the greatest challenges for plastic and reconstructive surgeons [1]. Currently, surgical options to restore bone and cartilage defects includes autologous grafts, causing donor site morbidity and are limited by the availability of suitable tissue [1]. Synthetic options available include inert materials such as porous polyethylene Medpor, which acts as a mechanical support for tissue ingrowth but does not allow for bone or cartilage regeneration [1]. Therefore, there is a clinical demand to create advanced materials, which can promote bone and cartilage formation [1].

Adipose derived stem cells (ADSCs) have demonstrated to be an exciting stem cell source for regenerative medicine due to their ease of isolation, high proliferative capacity and multi-differentiation potential [2], [3]. In order to be able to use this readily accessible source of stem cells for repairing skeletal tissue in the clinic, optimal conditions for their differentiation and maturation are needed. One approach to restoring craniofacial defects is to create biomaterials that can guide stem cell behavior towards bone and cartilage formation. It is well known that surface chemistry can affect stem cell adhesion, proliferation and differentiation, proving to be an important parameter when considering biomaterial fabrication. Previously, to evaluate the effect of surface chemistry on stem cell behavior, self-assembled monolayers have been utilised [2], [4], [5]. Curran et al. demonstrated that clean silane modified surfaces with NH_2_ surfaces promoted osteogenesis but not chondrogenesis of mesenchymal stem cells (MSCs) [4]. However, self assembled monolayers (SAMs) are limited to evaluating gold and glass substrates.

Plasma surface modification is an effective and economical technique, which can be used to modify the surface chemistry of biomaterials with various shapes and sizes and then study cellular interactions [6], [7]. The plasma process results in a physical and chemical modification of the surface of the biomaterial, while its bulk properties remain unchanged [7]. Plasma polymerisation allows the introduction of a wide range of surface chemistries and forms a layer of adherent functional groups on the biomaterial surface [7]. The process involves activating the surface with gases such as oxygen, nitrogen or argon and then depositing the desired functional groups onto the material surface [7].

We have previously demonstrated that our nanocomposite scaffold, which encompasses polyhedral oligomeric silsesquioxane (POSS) nanoparticles within a polyurethane backbone, can support the ADSC adhesion and growth *in vitro* [3]. We have previously shown that we can modify POSS-PCU with NH_2_ and COOH functionalisation using plasma polymerisation [8]. Allylamine and acrylic acid were used to deposit —NH_2_ and —COOH groups, respectively, on the nanocomposite scaffolds [8]. We also have some initial data that allylamine modification may increase osteogenic differentiation of ADSCs [9]. Here we tested the hypothesis that different modifications of the chemical groups on the surface of the nanocomposite polymer could increase adhesion of ADSCs to the nanocomposite scaffold and selectively enhance their differentiation towards bone or cartilage. We show here that by varying the chemical functionality on the surface of the nanocomposite scaffolds we can indeed enhance adhesion as well as preferentially stimulate human ADSC differentiation capability towards the chondrogenic or osteogenic lineage. These results demonstrate that plasma polymerisation of biomaterials can be a useful tool for improving the skeletal differentiation of ADSCs.

## 2 Materials and methods

All reagents and tissue culture plastic were from Sigma Alrich (UK) unless otherwise specified.

### 2.1 POSS-PCU nanocomposite synthesis and 3-dimensional (3D) scaffold fabrication

The nanocomposite polymer, POSS-PCU, was synthesised, as described previously [8]. Briefly, polycarbonate polyol (2000 mwt) and trans-cyclohexanechloroydrinisobutyl-silses-106 quioxane (Hybrid Plastics Inc) was placed into a 500 ml flask containing a mechanical stirrer and nitrogen inlet. The POSS cage was dissolved into the polyol solution using heat followed by cooling to 70 °C. At a temperature of 75–85 °C for 90 min flake 4,4′-methylenebis(phenyl 109 isocyanate) (MDI) was added to the polyol blend mixture to form a pre-polymer. To create a solution dimethylacetamide (DMAC) was then added slowly to the pre-polymer. Following cooling to 40 °C chain extension was then carried out by the addition of ethylenediamine and diethylamine in DMAC in a drop wise manner. This process created a POSS-modified polycarbonate urea-urethane in DMAC solution.

The POSS-PCU polymer was fabricated as a 3D scaffold using a coagulation technique. Firstly, sodium chloride (NaCl) was dissolved in 18% weight solution of POSS-PCU in DMAC containing Tween-20 surfactant. Stainless steel sieves (Fisher Scientific, Loughborough UK) were used to obtain a NaCl mixture of 200–250 μm size. The final solution was then dispersed and degassed in a Thinky AER 250 mixer (Intertronics, Kidlington, UK). A 1:1 wt ratio of NaCl to POSS-PCU was used in all experiments. The polymer mixture was then spread evenly onto circular stainless steel moulds and directly placed into deionised water for initially 30 h. Following this period, frequent water changes were carried out to dissolve out to remove the NaCl porogen particles and DMAC from the polymer solution for 7 days to create a porous scaffold. Then 8 cm × 8 cm circular polymer sheets with 700–800 μm thickness were manufactured. For cell culture analysis the circular sheets of polymer were cut into 16 mm diameter discs to be used in 24-well plates, using a steel manual shape cutter. Prior to cell seeding polymer discs were briefly sterilized using 70% ethanol and washed three times with sterile phosphate buffer (PBS) as previously reported [8]. They were then incubated in Dulbecco's Modified Eagle's Medium/Nutrient Mixture F-12 Ham (DMEM/F12) supplemented with 10% fetal bovine serum (FBS) and 1% antibiotic solution (penicillin) for 24 h prior to cell seeding.

### 2.2 POSS-PCU 3D scaffold surface modification with plasma

Plasma surface modification was carried out by using low (radio frequency) plasma generator operating at 40 kHz at 100 W. Scaffolds to be treated were placed in a 24 well plate [8]. Plasma modification was performed as a 2-stage procedure, surface activation and plasma polymerisation. Surface activation was achieved by exposing samples to oxygen plasma for 5 min, at 40 kHz. With gas flow rate of 0.4 mbar. Plasma polymerisation was carried out by introducing either allylamine or acrylic acid monomers (Sigma Aldrich, UK) at 0.4 mbar pressure for further 5 min at 100 W to produce NH2 and COOH scaffolds, respectively. Samples were immediately stored in a desiccator under vacuum until further use.

### 2.3 Bicinchoninic acid (BCA) assay protein quantification assay

Total serum protein adsorption on unmodified and modified samples was determined by using BCA assay (ThermoFisher Scientific) as described previously (n = 6) [8]. Briefly, scaffolds were incubated with complete growth medium at 37 °C for 1 h. The scaffolds were washed three times with phosphate buffer saline (PBS, pH = 7.4) before adding BCA reagent to each well and incubated at 37 °C. The absorbance was measured at 562 nm (Fluoroskan Ascent FL, Thermo Labsystems, UK). Scaffolds incubated in serum free medium were used as a control (n = 6).

### 2.4 Specific protein adsorption to plasma modified scaffolds: fibronectin and vitronectin

Specific protein adsorption to plasma-modified scaffolds was performed according to Seo et al. [10]. Each POSS-PCU scaffold was immersed in 0.5 ml of bovine fibronectin (bFN) or vitronectin (bVN) solution (10 μg/ml^−1^ in PBS, pH = 7.4) at 37 °C for 1 h prior to the biochemical evaluations. The bFN solution was then removed and the wells carefully washed with fresh PBS twice. After 0.5 ml of sodium dodecyl sulfate (SDS) (10 mg/ml) was added, each plate was sonicated for 20 min at room temperature. The protein concentration in the SDS solution of the bVN and bFN was then determined using a micro-BCA™ protein assay reagent kit and absorbance measured at 562 nm using Fluoroskan Ascent FL, (Thermo Labsystems, UK) (n = 6).

### 2.5 Functional presentation of cell-binding domains of adsorbed fibronectin and vitronectin

The accessibility of cell-binding domains of bovine fibronectin (bFN) and bovine vitronectin (bVN) adsorbed on POSS-PCU scaffolds and tissue culture plastic (TCP) was examined by enzyme-linked immunosorbent assay (ELISA) using monoclonal antibodies (mAbs) directed to epitopes of the RGD-containing domains as described previously [11]. For protein adsorption, the different substrates were incubated in the protein solutions (bFN or bVN) at different concentrations at 37 °C. After washing with PBS, all surfaces were blocked in 1% w/v BSA (bovine serum albumin/PBS) for 60 minutes at 37 °C, and then incubated with specific mouse monoclonal antibodies to bFN and bVN at pre-determined optimal concentrations (0.17 μg/ml anti-bFN and 1μg/ml anti-bVN; AntibodyChain, UK) in 1% w/v BSA/PBS, for 1 h at 37 °C. After washing three times in PBST (0.05% Tween-20 in PBS), POSS-PCU scaffolds were incubated in horseradish peroxidase-conjugated anti-mouse IgG (H+L) secondary antibody (Molecular Probes, 1:1000 in 1% w/v BSA/PBS) for 1 h at 37 °C. After three washes in PBST, samples were incubated in *o*-phenylenediamine dihydrochloride substrate (OPD, 0.4 mg/mL in 0.05 M phosphate–citrate buffer pH 5.0, with 0.012% v/v hydrogen peroxide) and colour was allowed to develop for 10 min at room temperature. Supernatants were transferred to new 96-well plates and absorbance measured at 450 nm. Controls (incubations without protein and/or without primary mAbs) were also assayed in parallel (n = 6).

### 2.6 Human adipose stem cell cultures

Human adipose tissue-derived stem cells (ADSC) were isolated according to the method described by Naderi et al. from fat [12]. Discarded adipose tissue was collected from adult female patients (n = 6, age range 31–55 years) undergoing abdominoplasty procedures. The North Scotland ethical review board, reference number 10/S0802/20, approved this study; all participants gave informed written consent. In brief, following removal of fibrous tissue and visible blood vessels, samples were cut into small pieces (<3 mm^3^) and digested in Dulbecco's Modified Eagle's Medium/Nutrient Mixture F-12 Ham (DMEM/F12) containing 300 U/ml crude collagenase I (Invitrogen, Life Technologies Ltd, Paisley, UK) for 30 min in a humidified incubator (37 °C, 5% CO_2_). Subsequently, 10% FBS was added to the dispersed material and filtered through 70 μm Cell Strainers (BD Biosciences, Oxford, UK). After centrifugation (290*g* for 5 min), the supernatant was removed and the ADSC-containing pellet re-suspended. The number of viable cells was determined by cell counting on a haemocytometer and trypan blue exclusion. Cells were cultured for up to 2 passages in DMEM/F12 supplemented with 10% FBS and 1% penicillin solution. At each subsequent passage, cells were seeded to sub-confluence in 75 cm^2^ culture flasks for 7–8 days at a cell density of 3 × 104 per cm^2^. When the cells reached approximately 80% confluence, subculture was performed through trypsinisation. The cell suspension was centrifuged (290*g* for 5 min), the pellet was re-suspended and cells were counted as before and then seeded on the polymer discs for analysis. ADSCs derived from the six donors (passage 2–4) were used as independent biological replicates.

### 2.7 Adipose stem cell differentiation

At day 0, scaffolds were placed in the bottom of the 24 well plate and incubated overnight with ADSC culture medium. At day 1, 10^5^ ADSCs were added to each scaffolds in fresh medium. This plating density was used in all experiments assessing cell behavior and differentiation unless otherwise specified. Once confluent on day 3 ADSCs were differentiated according to Guasti et al. [3]. In brief the following protocols were used.

#### 2.7.1 Chondrogenic differentiation – quantification

Confluent ADSCs were incubated in chondrogenic differentiation medium containing DMEM 10% FBS, 0.1 μM dexamethasone, 10 ng/ml transforming growth factor (TGF-β1) (R&D Systems, UK), insulin-transferrin-selenium (ITS) (Life Technologies), and 50 μg/ml ascorbate. Medium was changed every two days for 3 weeks. After 3 weeks, cells were either fixed in 4% PFA (paraformaldehyde) for immunocytochemistry or staining protocols, or RNA was extracted for RT-qPCR analysis, and the medium was taken for ELISA analysis for elastin and glycosaminoglycans. For staining, the scaffolds were further rinsed with 0.1 N HCl for 5 min and stained with Alcian Blue (1% in 0.1 N HCl). For quantification, the dye was extracted with 6 M guanidine hydrochloride overnight at room temperature, and absorbance measured at 595 nm. Fold changes were calculated, taking untreated controls as reference (n = 6). As a control, undifferentiated ADSCs were grown in the same 3D scaffolds in expansion medium.

#### 2.7.2 Osteogenic differentiation – quantification

Confluent ADSCs were incubated in osteogenic medium containing DMEM 10% FBS, 0.1 μM dexamethasone, 100 μg/ml ascorbate, and 10 mM β-glycerophosphate. The medium was changed every two days for 3 weeks. After 3 weeks, cells were fixed in 4% PFA for immunocytochemistry or RNA was extracted for RT-qPCR analysis and the medium was taken for ELISA analysis of osteocalcin and colorimetric analysis of collagen. For staining scaffolds were fixed in ice-cold 70% ethanol for 1 h, washed with H_2_O, and stained with 1% alizarin red. For quantification of staining, cells were incubated with 10% acetic acid for 30 min at room temperature, scraped, transferred to 1.5-ml vials, and heated at 85 °C for 10 min. Debris was eliminated by centrifugation and the supernatant absorbance at 405 nm determined. Fold changes were calculated, taking untreated controls as reference (n = 6). As a control, undifferentiated ADSCs were grown in similar 3D culture conditions in expansion medium.

### 2.8 F-Actin morphology staining

To stain ADSCs for actin 15,000 cells were seeded onto the scaffolds. The media was then removed from the 24-wells at 6 and 24 h. The cells were then washed with PBS several times and fixed with 4% (w/v) paraformaldehyde in PBS pre warmed at 37 °C for 10–15 min. Cells were then washed with 0.1% Tween 20 thrice, followed by incubation with 0.1% TritonX-100 for 5 min to improve permeability. Rhodamine-conjugated phalloidin (Thermo Fisher Scientific, UK) was then added in the ratio 1:40 (stock 1:1000 in methanol) in PBS and left for 40 min. Cells were then washed three times and then mounted onto slides with DAPI (4′,6-diamidino-2-phenylindole, 1:500) to stain the nuclei. The cells were then visualized and digitally scanned using a confocal laser-scanning microscope (LSM 710, Zeiss). The cells were analysed using ImageJ Software 1.48V (National Institute of Health USA) to determine cell circularity and cell area (the surface occupied by spread of the actin cytoskeleton). Cell circularity is used to provide a quantification of cell shape. The formula used for circularity index (CI) determination is CI = 4π^∗^(Area/Perimeter^2^). A value of 1.0 indicates a perfect circle and a value of 0.0 a totally elongated structure [13]. A total of 30 cells were analysed on 6 scaffolds, taking an average for comparison (n = 6). Cell attachment was assessed by dividing the total cell coverage by the scaffold surface area and expressing it as a percentage (n = 6).

### 2.9 Metabolic activity – Alamar blue™ assay

Scaffolds were placed in 24 well plates and sterilized as explained previously prior to seeding with 25,000 ADSCs per scaffold. Cellularized scaffolds were cultured for 24 h and then moved to fresh wells prior to assay to only assay cells attached to the scaffolds. ADSC cytotoxicity and viability was assessed using the commercially available assay Alamar blue™ (Life Technologies, UK) according to the manufacturer’s instructions on days 1, 2, 4, 7, 14 and 21. Briefly, after 4 h of incubation with Alamar blue dye, 100 μl of media was placed into 96 well plates and fluorescence was measured at excitation and emission wavelength of 530 and 620 nm using Fluoroskan Ascent FL, (Thermo Labsystems, UK). As this assay is non-toxic to cells, the same set of scaffolds were used for further testing by washing them with PBS and then adding fresh cell culture media (n = 6).

### 2.10 Analysis of cell proliferation using DNA quantification

To assess ADSC cells proliferation a Fluorescence Hoechst DNA Quantification Kit was utilized to quantify the DNA content on the POSS-PCU scaffolds at days 1, 2, 4, 7 and 14. Scaffolds were seeded with 50,000 ADSCs for analysis (n = 6). In preparation for analysis all samples were put in −80 °C at the specific time points prior to removal of the media sample and washing with PBS three times, with the addition of 500 μl of deionised water two days later to each 24-well. At day 16 all polymer samples were analysed. The cell samples underwent three free-thaw cycles. A standard curve was first composed using known quantities of DNA (0, 0.15625, 0.3125, 0.625, 1.25, 2.5, 5 μg/ml). To begin 500 μl of Hoechst 33,258 (10 mg/ml) was added to 500 μl of fluorescence assay buffer (100 nM Tris-HCl, pH 7.4, with 10 nM EDTA and 2 M NaCl) with 4.5 ml of deionised water to make the Hoechst diluted solution. Then 100 μl of each cell sample and 100 μl of Hoechst diluted solution was added to 96-well dark plate. After 5 min at room temperature in the dark, the fluorescence was measured with excitation set at 360 nm and emission at 460 nm using the Anthos 2020 microplate reader (Biochrome Ltd, UK).

### 2.11 Quantification of extracellular matrix (ECM) proteins

Secretion of extracellular matrix components, elastin (Biocolour Fastin Elastin Assay) and osteocalcin (R&D), by ADSCs into the culture medium was assessed at 14 and 21 days. Collagen was detected using two assays, namely the Pico Sirius Red (PSR) method and hydroxyproline quantification.

(A) Total collagen expression was analysed at day 21 using the PSR method as described previously [14]. Briefly, cells were first fixed in methanol overnight at −20 °C. After washing with PBS they were stained at room temperature for 4 h with the PSR stain (0.1%). Excess dye was washed with PBS three times and 0.1% acetic acid. The stained cells were then dried for spectrophotometric analysis. The PSR solution was eluted in 200 μl of 0.1 N sodium hydroxide per well. The solution was placed on a rocker at room temperature for 1 h before the absorbance was read at 540 nm with the Anthos 2020 micro plate reader (Biochrome Ltd, UK). A reference standard was prepared using 1, 5, 10, 20, 30, 40 and 50 μg of bovine collagen dissolved in deionised water prior to analysis of the polymer scaffolds (n = 6).
(B) Hydroxyproline content at 21 days were measured using a QuickZyme hydroxyproline assay (2B Scientific UK) according to the manufacturer’s instructions (n = 6). In brief, scaffolds underwent acid hydrolysis with 6 M HCl and then hydroxyproline content was measured using a standard curve with the absorbance measured at 570 nm.

### 2.12 Alkaline phosphatase assay

The colorimetric alkaline phosphatase (ALP) assay kit (Abcam) was used to assess ALP activity in ADSCs seeded on the scaffolds in osteogenic differentiation medium after 14 and 21 days. Staining was performed according to the manufacturer’s instructions (n = 6). In brief, 80 μl of supernatant was collected and then 50 μl of the p-nitrophenyl phosphate (pNPP) was added to the samples as a substrate followed by the addition of ALP enzyme solution for 60 min at 25 °C. The reaction was then stopped by adding 20 μl of Stop Solution and the absorbance was read at 450 nm (n = 6).

### 2.13 Analysis of extracellular matrix (ECM) and adhesion-related proteins by immunocytochemistry

After 21 days in culture, the cellularised scaffolds were washed in PBS and fixed in 4% PFA overnight at 4 °C. The scaffolds were then washed in PBS three times and then embedded in Optimal Cutting Temperature (OCT) and cryosectioned (40 μm thick). Polymer sections were then permeabilised (0.5% Triton X-100 in PBS) and blocked in 0.5% BSA in PBS for 1 h at room temperature. Sections were incubated with primary antibodies diluted in blocking solution overnight at 4 °C (Supplementary data Table 1). After several washes with PBS, sections were incubated with secondary antibodies for 2 h at room temperature. Cell nuclei were visualized with Hoechst 33,258 (2.5 μg/ml final concentration). Stained sections were then visualized and digitally scanned using a confocal laser-scanning microscope (LSM 710, Zeiss).

### 2.14 Scanning Electron Microscopy (SEM)

For cell morphology analysis, the discs were fixed with 2.5% w/v glutaraldehyde/PBS for 48 h as described previously [8]. The scaffolds were then dehydrated using a series of acetone alcohol solutions (distilled water, 50%, 70%, 90%, 100%, 100%) at room temperature and then CO_2_ critically point dried. The polymer disc scaffolds were then attached to aluminum stubs with double sided sticky tabs before being coated with gold using a sc500 (EMScope) sputter coater. The polymer discs were then analysed and photographed using the FEI Quanta 200F Scanning Electron Microscope.

### 2.15 Expression of extracellular matrix and adhesion-related genes by RT-qPCR

RNA was extracted from cellularised scaffolds using Tri-Reagent (Life Technologies) according to the manufacturer's instructions 21 days after seeding the ADSCs. RNA was reverse-transcribed with Moloney murine leukemia virus reverse transcriptase (Promega, Madison, WI). Primer sequences and annealing temperatures for each set of primers are shown in Supplementary Table 2. Real time quantitative polymerase chain reaction (qPCR) was preformed with an ABI Prism 7500 sequence detection system (Applied Biosystems) using the QuantiTect SYBR Green PCR kit (Qiagen, Hilden, Germany) according to the manufacturer's instructions. Gene expression data were normalized using GAPDH housekeeping gene as a reference using the 2−ΔΔCt method.

### 2.16 Chorioallantoic membrane (CAM) grafting

CAM grafting was carried out as described previously by Guasti et al. [3]. In brief, fertilized Brown Leghorn eggs (Needle Farm) were incubated at 38 °C in a humidified forced flow incubator. All procedures were carried out under the Animals Scientific Procedures Act 1986. On day 3 of incubation, 2 ml of albumin was removed to detach the developing chorioallantoic membrane (CAM) from the shell; the eggs were then windowed and sealed with adhesive tape. On day 7 of incubation, the tape was removed and the CAM was slightly scratched with forceps to induce a small amount of bleeding. Small pieces (approximately 2 × 2 mm) of POSS-PCU seeded with or without hADSCs were placed on top of the scratched area of the CAM (6 eggs per group). The eggs were then sealed with adhesive tape and returned to the incubator for additional 7 days of incubation. The scaffolds were then photographed *in ovo* at day 14 of incubation, before overnight fixation in 4% PFA. The scaffolds were then washed in PBS, embedded in OCT, sectioned and processed for immunocytochemistry as described above.

### 2.17 Statistical analysis

Statistical analysis of the results was performed using Graph Pad (Prism. Statistical significance was calculated by two-way and one-way ANOVA, with Tukey HSD post hoc analysis where P *<* 0.05 value was considered statistically significant.

## 3 Results

### 3.1 Analysis of protein adsorption

**Fig. 1:**
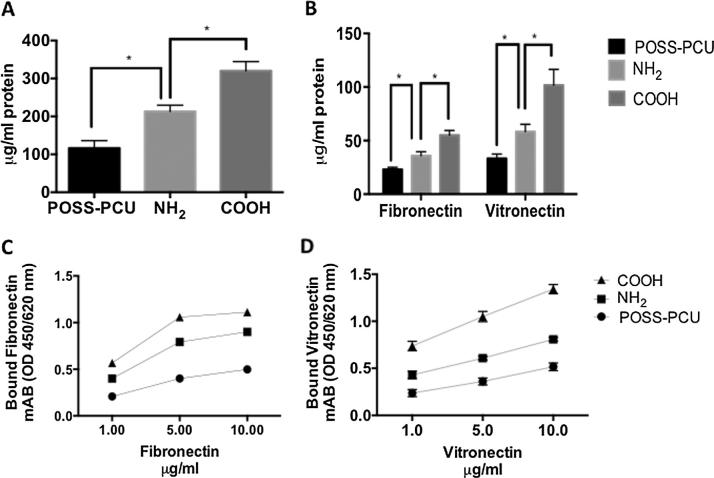
Protein adsorption on plasma modified scaffolds. A) Serum protein detection by BSA assay; note that after 1 h incubation a greater amount of protein is adsorbed on COOH than on NH_2_ scaffolds (p < 0.05). B) Fibronectin and vitronectin adsorption detected by election of both proteins’ adsorption is greater on COOH than NH_2_ scaffolds (p < 0.05). C) Fibronectin and D) vitronectin conformation detected using monoclonal antibodies is also greater on COOH than NH_2_ scaffolds and unmodified scaffolds (p < 0.05).

Adsorption of proteins onto a surface dictates cell adhesion to a biomaterial surface [15]. Protein adsorption was evaluated after plasma surface modifications (Fig. 1). After 1 h, serum protein adsorption was significantly greater on the COOH than NH_2_ scaffolds as indicated by the BCA assay (p < 0.05) (Fig. 1A). Levels of fibronectin (FN) and vitronectin (VN) were also greater on the COOH surfaces compared to the NH_2_ surfaces (Fig. 1B). The conformation of fibronectin (Fig. 1C) and vitronectin (Fig.1D) proteins assessed using monoclonal antibodies was also greater on the COOH modified surfaces compared to the NH_2_ surfaces and unmodified surfaces (p < 0.05).

### 3.2 Assessment of ADSC adhesion

**Fig. 2:**
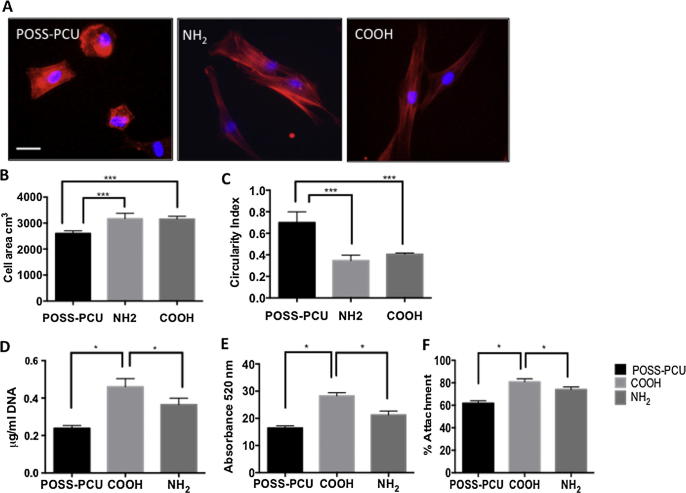
Actin staining of adipose-derived stem cell (ADSC) spreading and metabolic activity on plasma modified scaffolds. A) Detection of actin by phalloidin staining (red) in ADSCs 6 h after seeding on control and modified POSS-PCU scaffolds; note that the actin cytoskeleton of cells on plasma modified scaffolds is more elongated than in cells on unmodified POSS-PCU scaffolds. Nuclei are stained with Dapi (blue). Scale Bar: 20 μm. B–C) Measure of actin-covered area (B) and circularity index (C) of ADSCs on the different scaffolds at 24 h; note significantly increased “actin” area and reduced circularity index on plasma modified scaffolds. D-F) Assessment of DNA content (D), cell metabolic activity (E) and percentage cell attachment (F) in ADSC at 24 h. Note that DNA content and cell metabolic activity are significantly greater on the COOH modified scaffolds. ^∗^p < 0.05, ^∗∗^p < 0.01, ^∗∗∗^p < 0.001.

**Fig. 3:**
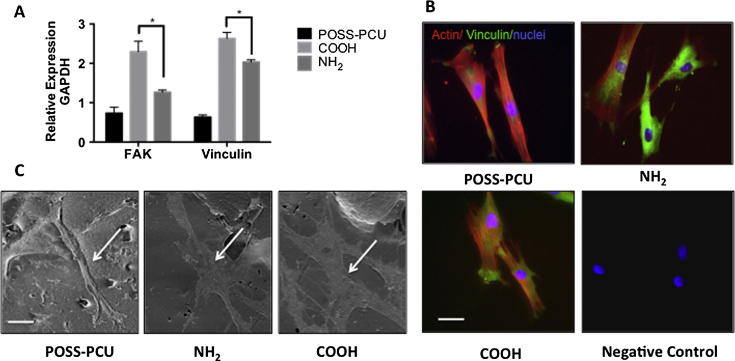
Adhesion of adipose-derived stem cells (ADSCs) on plasma modified scaffolds 24 h after seeding. A) Expression of focal adhesion kinase (FAK) and vinculin assessed by RT-qPCR. Note greater expression of these transcripts on the COOH modified scaffolds (p < 0.05). B) Double staining for vinculin (green) and actin (red); note expression of vinculin in all scaffolds. Nuclei are stained with Dapi (blue). Scale bar: 20 μm. C Scanning electron microscopy images of ADSCs on the different scaffolds; the arrows point to ADSCs spread on the scaffolds Scale bar: 30 μm.

As the morphology of ADSCs seeded on POSS-PCU cannot be easily imaged, the actin cytoskeleton was stained with phalloidin to obtain information on cell attachment and spreading on the different scaffolds. At 6 h, the actin cytoskeleton of ADSCs plated on the COOH and NH_2_ scaffolds appeared to be more elongated than that of cells on the POSS-PCU scaffolds (Fig.2A). The actin-covered area was significantly greater and with a lower circularity index in cells on the COOH and NH_2_ scaffolds than on the POSS-PCU scaffolds (Fig.2A-C). At 24 h, DNA content (Fig. 2D) and viability, assessed by the alamar blue assay (Fig. 2E-F), of ADSCs are significantly higher on plasma surface modified scaffolds suggesting enhanced cell adhesion (p < 0.05). However, COOH scaffolds promoted cell adhesion to a greater degree than NH_2_ scaffolds (p < 0.05). By 24 h the ADSCs had a stretched morphology on COOH, NH_2_ and unmodified scaffolds (Fig. 3).

Vinculin and FAK are important for cell-matrix adhesion [16]. Analysis of vinculin and FAK expression of the ADSCs by RT-qPCR after 24 h on the plasma modified scaffolds showed significantly increased levels of these transcripts compared to the unmodified scaffolds (p < 0.05) (Fig. 3A). Furthermore, expression of vinculin and FAK mRNA was greater on COOH than NH_2_ scaffolds (p < 0.05). Double-staining for vinculin and actin at 24 h showed increased ADSC spreading on all surfaces as compared to 6 h (Fig. 2A) but higher expression of vinculin on modified scaffolds and differences in the actin cytoskeleton organisation (Fig. 3B); vinculin appeared to cluster more at the cell periphery in COOH than in NH_2_ scaffolds. More extensive ADSC spreading on the modified scaffolds was further supported by SEM analysis (Fig. 3C).

### 3.3 Analysis of ADSC proliferation

**Fig. 4:**
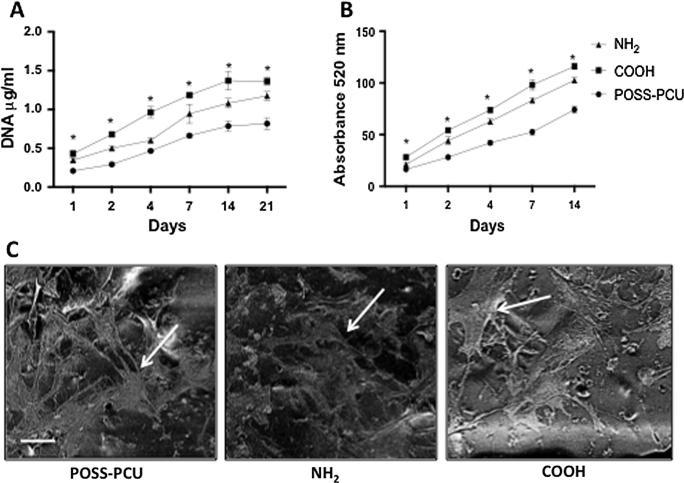
Proliferation of adipose-derived stem cells (ADSCs) on plasma modified scaffolds. Long-term culture of the ADSCs on the plasma modified scaffolds. A) DNA assay and B) Alamar blue assay confirmed the long-term culture of ADSCs on plasma-modified scaffolds over 21 days was the greatest on COOH scaffolds compared to NH_2_ and unmodified scaffolds (^∗^p < 0.05). C) Scanning Electron Microscopy (SEM) confirmed the stretched morphology of the ADSCs after 14 days of culture on the plasma-modified scaffolds. Arrows point to ADSCs on scaffolds. Scale bar: 20 μm.

The viability and cell number of the ADSCs was compared over 21 days on the plasma-modified scaffolds. DNA assay and cell viability assay demonstrated that more ADSCs were present on COOH scaffolds than on NH_2_ and unmodified scaffolds at all time-points examined (p < 0.05) (Fig.4A-B). SEM demonstrated that the ADSCs adopted a spindle-like morphology after 14 days of culture on surface modified scaffolds that was comparable to that of ADSCs grown in unmodified scaffolds (Fig.4C).

### 3.4 ADSC osteogenic and chondrogenic differentiation

**Fig. 5:**
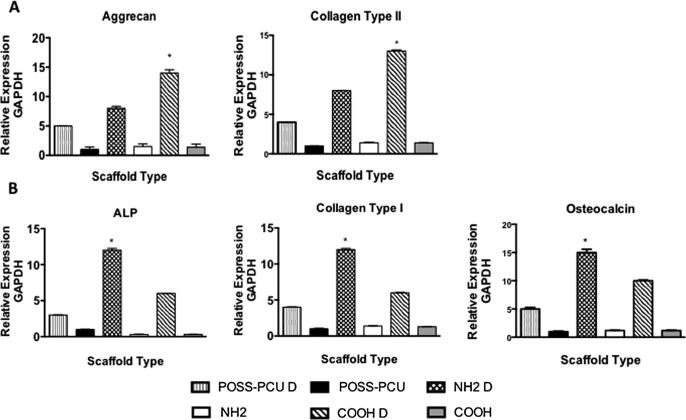
Expression of chondrogenic and osteogenic differentiation markers in adipose-derived stem cells (ADSCs) grown in control and differentiation (indicated by “D”) media on plasma modified scaffolds. Gene expression assessed by RT-qPCR at 21 days. of culture is shown. A) Expression of aggrecan and collagen type II following ADSC chondrogenic differentiation; upon differentiation both transcripts are significantly higher on the COOH scaffolds than on the other scaffolds. B) Expression of alkaline phosphatase (ALP), collagen type I and osteocalcin following ADSC osteogenic differentiation; upon differentiation gene expression increase is greater on NH_2_ modified scaffolds than on the other scaffolds (^∗^p < 0.05).

ADSC were differentiated along the osteogenic and chondrogenic lineages on the plasma modified scaffolds as described by Guasti et al. 2012 [3]. The scaffolds were manufactured to be porous structures to allow tissue ingrowth. The mRNA expression of differentiation specific markers was determined at 3 weeks. The greater up-regulation of chondrogenic marker expression, aggrecan and collagen II, was observed on the COOH scaffolds (p < 0.05) (Fig. 5). In contrast, osteogenic marker transcripts, ALP, collagen type I, and osteocalcin were more extensively up-regulated on the —NH_2_ scaffolds (p < 0.05).

**Fig. 6:**
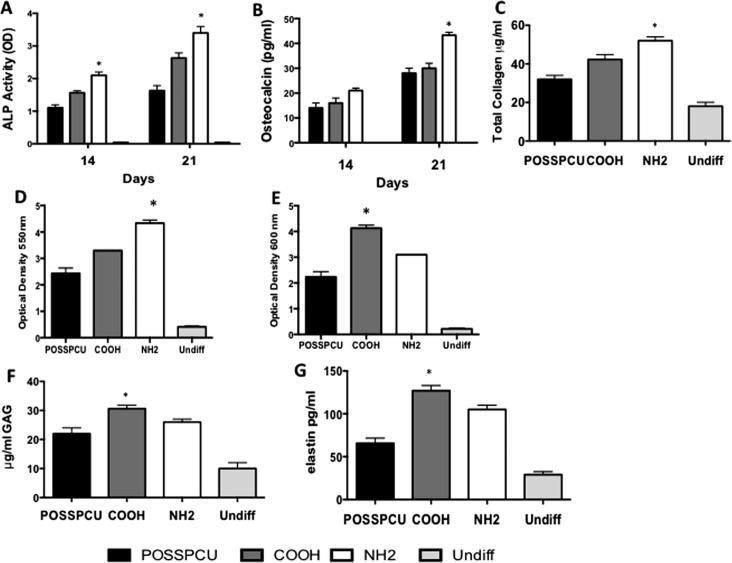
Analysis of protein secretion by the adipose-derived stem cells (ADSCs) on plasma-modified scaffolds. A-B) Alkaline phosphatase (ALP) activity and osteocalcin production following 14 and 21 days of osteogenic differentiation; both are enhanced on NH_2_ scaffolds. C) Total collagen secretion after 21 days was greater on the NH_2_ surfaces than COOH surfaces. D) Quantification of the Alizarin Red Staining was significantly greater on the NH_2_ scaffolds than on all other scaffolds (^∗^p < 0.05 E) Quantification of Alician Staining was significantly greater on the COOH scaffolds than on all other scaffolds F-G) Glycosaminoglycans (GAG) and elastin detection following 21 days of chondrogenic differentiation; the higher levels are detected on COOH scaffolds (^∗^p < 0.05). G) (^∗^p < 0.05).

**Fig. 7:**
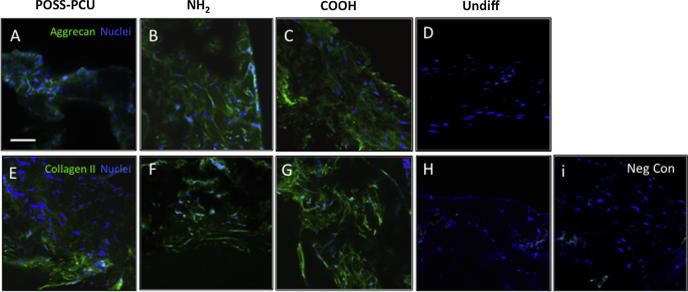
Matrix deposition by adipose-derived stem cells (ADSCs) on plasma modified scaffolds after 3 weeks of chondrogenic differentiation. A-H) Detection of aggrecan (A-D) and collagen type II (E-H) expression (both green) by immunocytochemistry. Negligible levels of expression are detected in undifferentiated controls (H). Nuclei are in blue (DAPI staining). Scale bar: 250 μm. I) Negative control where primary antibody was omitted.

**Fig. 8:**
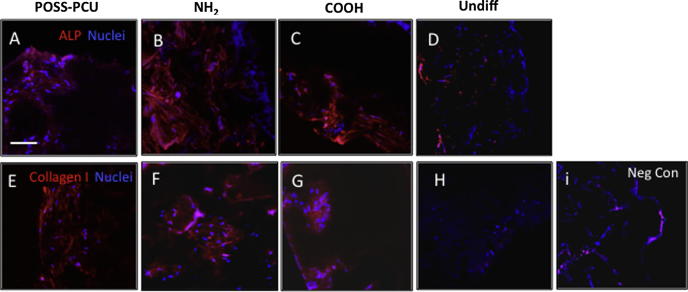
Matrix deposition by adipose-derived stem cells (ADSCs) on plasma modified scaffolds after 3 weeks of osteogenic differentiation on plasma modified scaffolds. A-H) Detection of alkaline phosphatase (ALP, A-D) and collagen type I expression (both red) by immunocytochemistry. Nuclei are in blue (DAPI staining). Note that staining levels of both proteins appear to be greater on NH_2_ than on COOH-modified and POSS-PCU scaffolds Negligible levels of expression are detected in undifferentiated controls (H). Scale bar: 250 μm. I) Negative control where primary antibody was omitted.

Occurrence of differentiation was also assessed at the protein level. Increased secretion of GAG and elastin in COOH scaffolds at 14 and 21 days of chondrogenic differentiation, and increased alician blue staining at 21 days were consistent with enhanced chondrogenesis in these scaffolds (Fig.6D-F). We also visualized aggrecan and collagen type II deposition by immunocytochemistry after 3 weeks of differentiation. While quantification of the immunostaining in scaffolds is difficult, there was apparent up-regulation of aggrecan and collagen type II (Fig. 7A-I) on COOH scaffolds, consistent with the increased mRNA levels (Fig. 5A). Chondroid matrix deposition was also demonstrated by SEM in all scaffolds (Fig. Supplementary Data 1A–C). In contrast, following 21 days of osteogenic differentiation, calcium deposition, ALP activity, osteocalcin and collagen secretion was significantly greater on NH_2_ scaffolds than COOH and unmodified scaffolds (p < 0.05) (Fig. 6A-C). Immunostaining for osteocalcin and collagen type I also suggested a preferential increase in these protein deposition in NH_2_ scaffolds (Fig. 8A-I). Deposition of osteoid matrix was also observed by SEM (Fig. Supplementary Data 1D–F). No mRNA and protein up-regulation was observed in ADSCs in scaffolds without differentiation medium, demonstrating that while specific chemistry (NH_2_ and COOH) enhances ADSC differentiation, growth factors are required to induce tissue specific differentiation of these cells.

### 3.5 Vascular response to ADSC-POSS-PCU bioscaffolds

**Fig. 9:**
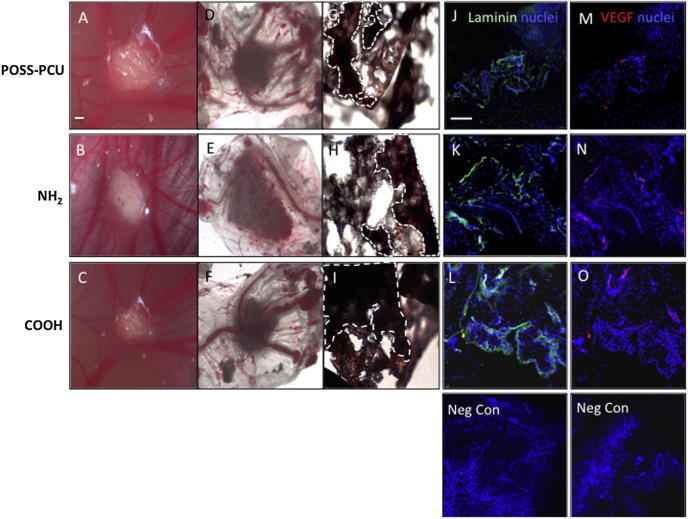
Chorioallantoic membrane (CAM) grafting of adipose-derived stem cells (ADSCs) on plasma modified scaffolds for 7 days. A-C) Images of CAM-grafted scaffolds *in vivo* and D-F) after removal from the CAM. G-I). Scaffold sections stained with hematoxylin and eosin. Dotted lines indicate the edge of the scaffold material. Scale bar: 500 μm. J-L) Detection of laminin (green, J-L) and vascular endothelial growth factor (VEGF) (red, M-O) and by immunocytochemistry in scaffold sections. Staining levels in POSS-PCU scaffolds appear to be lower than in NH_2_ and COOH scaffolds. Scale bar: 200 μm.

It is important to establish whether surface chemistry not only can enhance stem cell differentiation, but also support angiogenesis and tissue ingrowth to allow for successful implantation *in vivo* (Fig. 9). CAM grafting experiments (6 scaffolds) showed cell ingrowth and vessel formation in all scaffolds by 7 days both by gross morphology (Fig.9A-I) and expression of blood vessel markers, VEGF and laminin by immunohistochemistry (Fig.9J-Q). While accurate quantification of the immunofluorescence in the 3D scaffold was difficult, visual analysis of six sections per scaffold (n = 6) suggested a similar level of VEGF and laminin expression on the NH_2_ and COOH scaffolds, and that this was greater than on unmodified scaffolds.

## 4 Discussion

This study shows for the first time, that plasma polymerisation of POSS-PCU nanocomposite scaffolds with NH_2_ and COOH groups selectively enhance differentiation of human ADSC along the osteogenic and chondrogenic lineages, respectively. Furthermore, these modifications increase ADSC adhesion to the nanoscaffold and their proliferation, as well as supporting angiogenesis, as suggested by the *in vivo* CAM-grafting model.

### 4.1 Surface chemistry modulates ADSC adhesion and growth

We have shown enhanced protein adsorption on both NH_2_ and COOH functionalized scaffolds, with the greater effect observed on COOH scaffolds. Surface properties of a biomaterial affect the extent of protein adsorption, denaturation and epitope exposure [15]. Furthermore, it is well established that the degree of cell adhesion to synthetic surfaces arises from differences in protein adsorption [15].

Cell adhesion serum proteins, such as fibronectin and vitronectin play a vital role in cell adhesion to an artificial material. As well as protein quantity, the availability of specific protein binding sites is crucial in determining cell adhesion. Enhanced protein adsorption and adsorption of vitronectin and fibronectin in optimal conformations on modified scaffolds may account for the increased ADSC adhesion we observed. The amine group (NH_2_) provides a positive charge to the biomaterial surface [15]. Fibrinogen has been shown to form hydrogen bonds with NH_2_ groups and adhere to the surface [15]. Amine has also been shown to promote the exposure of high-density receptors as well as focal adhesion components allowing for adhesion. Materials bearing COOH chemical groups displays a negative charged functionality to the surface [15].

Focal adhesion kinase (FAK) localises to focal adhesions to activate several signaling pathways including cell migration, proliferation and differentiation [15], [16]. Surface chemistry has also been shown to influence focal adhesion assembly and downstream cell signaling [17]. COOH surfaces were found to increase cell growth through up-regulation of focal adhesion components [17]. Vinculin is a key protein formed within focal adhesion complexes responsible for the connection between the integrin adhesion molecules and the actin cytoskeleton [18]. Hence the greater expression of FAK and vinculin in ADSCs seeded on the COOH than on NH_2_ modified scaffolds reported here, is consistent with greater ADSC adhesion on the COOH surface. Cell viability and DNA content over 14 days was also greater on the COOH modified scaffolds than on NH_2_ modified and unmodified scaffolds, though also NH_2_ functionalisation increased cell growth as compared to unmodified scaffolds. The greater protein adsorption leading to enhanced ADSC adhesion on COOH modified scaffolds is likely to have led to the greater cell number observed on this scaffold over time. Together, whereas human fibroblasts display similar behavior on NH_2_ and COOH functionalised scaffolds [8], ADSC adhesion and proliferation is increased on the latter. This suggests that the response to surface modification is cell type-dependent and highlights the importance of identifying the optimal modification for different cell types.

Further research using different biomaterials and a more comprehensive understanding of surface functionality and protein interactions will be required to elucidate the basis of the differences in ADSC adhesion observed with different plasma modifications.

### 4.2 Specific surface chemistry enhances osteogenic and chondrogenic differentiation and Maintains biocompatibility

The ultimate aim of this study was to evaluate if surface chemistry could be used to direct ADSC differentiation on nanocomposite scaffolds. Taking into consideration biochemical analysis, gene and protein evaluation, NH_2_ modification was found to provide an optimal environment to support osteogenesis, while COOH functionalisation promoted chondrogenesis.

Our findings that ADSC osteogenesis is supported by NH_2_ functionalisation is consistent with the work by Guo et al. where occurrence of osteogenesis of bone marrow-derived mesenchymal stem cells (BM-MSCs) on allylamine plasma modified surfaces in the presence of dexamethasone was demonstrated [19]. However, that study did not clearly show increased osteogenesis on the modified surfaces, as, unlike in our study, quantification of differentiation on multiple samples was not reported [19]. Liu et al. also studied allylamine plasma coatings and suggested that this modification promotes attachment, spreading, proliferation and osteogenic differentiation of human ADSCs [20]. A study by Chen’s et al. also indicated that NH_2_ functional groups support osteogenesis of amniotic membrane-derived MSCs on PCL (poly(ε-caprolactone)) surfaces [21]. On the other hand, Wang et al. found that acrylic acid with COOH functional groups supported osteogenic differentiation of rat BM-MSCs as indicated by calcium deposition and colony formation [22]. However, while we compared human ADSC behavior on both modifications, no direct comparison of the behavior of rat MSCs on COOH and NH_2_ functional groups was carried out in previous studies [9], [22]. We indeed observed increased osteogenesis of ADSCs on COOH modified scaffolds as compared to control ones, though to a significantly lesser extent than on NH_2_ scaffolds.

There have been no studies investigating the effect of surface chemistry on chondrogenesis of ADSCs and just a few looking at its effects on other MSCs. Using silane modified glass surfaces, Curran et al. demonstrated some evidence that COOH groups promoted chondrogenesis of human BM-MSCs by the upregulation of collagen II expression [5]. Functionalisation of carbon nanotubes with COOH was also shown to enhance bovine chondrocytes’ ability to express the chondrogenic marker, collagen II [23]. In contrast, Chen et al. reported that CH_3,_ rather than NH_2_ and COOH functional groups, promoted chondrogenesis of human amniotic membrane-derived MSCs [21].

The ability of tissue engineered constructs to support tissue ingrowth and vascularisation is vital to ensure the success of an implanted construct. We have shown that all functionalised scaffolds with ADSCs allowed for tissue ingrowth and supported host vessel ingrowth after 7 days *in ovo.* This is consistent with our previous study demonstrating that functionalisation of POSS-PCU with either NH_2_ or COOH promoted its vascularisation *in vivo* [8] and indicates that addition of ADSCs to the functionalised surfaces does not prevent their vascularization.

Understanding the interaction of stem cells with biomaterials is of utmost importance for the advancement of tissue engineering for restoring damaged or missing tissues [15]. This study has shown that surface chemistry modifications are an important tool for modulating ADSC differentiation. Future work will be aimed at understanding how NH_2_ and COOH affect ADSC chondrogenesis and osteogenesis pathway, as this was beyond the scope of the current study. The repair of critical size defects in long term *in vivo* studies will also need to be performed to fully understand the modified scaffolds ability to maintain the differentiated phenotype of the ADSCs. The use of plasma polymerisation has the potential to functionalise other biomaterial surfaces, including metals and ceramics. However, this will require a detailed investigation to determine the stability of chemical groups on different surfaces and optimize the plasma modification process for each individual biomaterial [24].

## 5 Conclusions

In conclusion, this study has important implications for skeletal tissue engineering using ADSCs, having established that NH_2_ modified scaffolds preferentially promote osteogenesis and COOH surfaces chondrogenesis of these cells. Previous evidence of no changes in the mechanical properties of POSS-PCU scaffolds following plasma functionalisation [8] together with the ability of these modified scaffolds to selectively enhance differentiation of ADSCs towards the desired phenotypes, and their biocompatibility supported by the short term *in ovo* studies, provide a strong basis for future testing in larger animal studies. Clinical application of plasma polymerisation for the generation of bone and cartilage constructs appears promising.

## Funding

This study was funded by the Medical Research Council (MRC) and Action Medical Research (AMR), Grant No. GN2239.

## Figures and Tables

**Supplementary Fig. 1:**
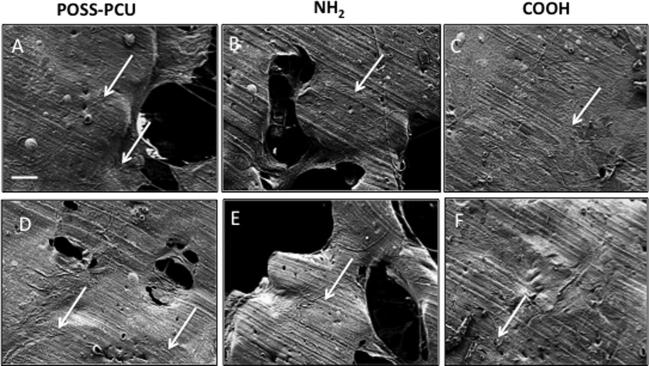
Scanning electron microscopy of the scaffolds after chondrogenic and osteogenic differentiation. Note extracellular matrix (ECM) formation on all scaffolds; arrows point to the edge of the ECM. A) Unmodified scaffolds. B) NH_2_ modified scaffolds. C) COOH modified scaffolds in chondrogenic differentiation conditions. D) Unmodified scaffolds. E) NH_2_ modified scaffolds. F) COOH modified scaffolds in osteogenic differentiation conditions. Scale bar in A: 200 μm; all images are at the same magnification.
