# Supplementary material for: Chemical group-dependent plasma polymerisation preferentially directs adipose stem cell differentiation towards osteogenic or chondrogenic lineages
Source: Acta Biomater. 2017 Mar 1;50:450–61. doi: 10.1016/j.actbio.2016.12.016 (PMC5331891; doi:10.1016/j.actbio.2016.12.016)
Supplement: Supplementary data 1 [file mmc1.docx]

**Supplemental Table 1**

**Primary and secondary antibodies used in this study.**

| **Antibody** | **Species** | **Types** | **Dilution** | **Incubation** | **Source** |
| --- | --- | --- | --- | --- | --- |
| **Primary** | | | | | |
| Collagen II | Rabbit | IgG | 1:100 | Overnight 4 ^O^C | Abcam |
| Collagen I | Mouse | IgG | 1:500 | Overnight 4 ^O^C | Millipore |
| Alkaline Phosphatase | Mouse | IgG | 1:1000 | Overnight 4 ^O^C | Abcam |
| Aggrecan | Rabbit | IgG | 1:500 | Overnight 4 ^O^C | Abcam |
| Vascular Endothelial  Growth Factor | Mouse | IgG | 1:100 | Overnight 4 ^O^C | Abcam |
| Laminin | Mouse | 1gG | 1:100 | Overnight 4 ^O^C | Millipore |
| Vinculin | Rabbit | 1gG | 1:250 | Overnight 4 ^O^C | Abcam |
| **Secondary** | | | | | |
| anti-mouse IgG Alexa Fluor^®^ 568 | Goat | IgG | 1:500 | 2 hours Room Temp | Molecular probe, Invitrogen |
| anti-rabbit IgG Alexa Fluor^®^ 488 | Goat | IgG | 1:500 | 2 hours Room Temp | Molecular probe, Invitrogen |

**Supplemental Table 2.**

**Primers used for QT-PCR**

| **Gene** | **Primers (5’-3’)** | **Annealing Temp. (C)** | **Cycles**  **(RT-PCR)** |
| --- | --- | --- | --- |
| Aggrecan | For TACTCTGGGTTTTCGTGACTC  Rev CGATGCCTTTCACCACGACTT | 56 | 32 |
| ALP^a^ | For CACGGGCACCATGAAGGAAAAG  Rev TGGCGCAGGGGCACAGGAGACT | 59 | 32 |
| Collagen I | For ATGCCTGGTGAACGTGGT  Rev AGGAGAGCCATCAGCACCT | 56 | 26 |
| Collagen II^a^ | For GCCTGGTGTCATGGGTTT  Rev GTCCCTTCTCACCAGCTTTG | 56 | 35 |
| Gapdh^a^ | For TGATGACATCAAGAAGGTGGTGAAG  Rev TCCTTGGAGGCCATGTGGGCCAT | 56 | 28 |
| Osteocalcin^a^ | For ACACTCCTCGCCCTATTG  Rev GATGTGGTCAGCCAACTC | 56 | 35 |
| Vinculin | For CCCCTGACATGGAAGACGATT  Rev TGTCATTGCCCTTACTAGACCAC | 59 | 35 |
| Focal Adhesion Kinase | For CAGGGTCCGATTGGAAACCA  Rev AAGCTTGACACCCTCGTTGT | 59 | 35 |

^a^ These primers detect both A and B isoforms.
